# Correlation between preoperative factors and final visual acuity after successful rhegmatogenous retinal reattachment

**DOI:** 10.1038/s41598-019-39839-2

**Published:** 2019-03-01

**Authors:** Norifumi Hirata, Takeshi Iwase, Misato Kobayashi, Kentaro Yamamoto, Eimei Ra, Hiroko Terasaki

**Affiliations:** Department of Ophthalmology, Nagoya University Graduate School of Medicine, Nagoya, Japan

## Abstract

We evaluated the preoperative optical coherence tomographic (OCT) findings in eyes with macula-off rhegmatogenous retinal detachment (RRD) and determined the factors that were significantly correlated with the postoperative best-corrected visual acuity (BCVA). The length of the preoperative photoreceptors was defined as the distance between the external limiting membrane (ELM) and the outer end of the outer segments of the photoreceptors in the OCT images. The mean length of the photoreceptors was 102.8 ± 28.7 µm with a range of 20 to 159 µm in eyes with RRD. The length of the preoperative photoreceptors was not significantly correlated with the preoperative BCVA but it was significantly correlated with the postoperative BCVA (*r* = −0.353, *P* = 0.003). Multivariate regression analyses revealed that the length of the photoreceptors (*β* = −0.388, *P* = 0.001) and the preoperative BCVA (*β* = 0.274, *P* = 0.021) were the only independent factors that were significantly associated with the postoperative BCVA. The length of the preoperative photoreceptors was significantly correlated with the postoperative photoreceptor length (*r* = 0.486, *P* < 0.001). Longer preoperative photoreceptors were significantly correlated with longer postoperative photoreceptors and better BCVA after successful reattachment. These results suggest that the preoperative length of the photoreceptors can be good factor to use for predicting the final BCVA following successful reattachment of macula-off RRD.

## Introduction

A rhegmatogenous retinal detachment (RRD) is a common cause of visual impairments, and a surgical reattachment of the retina is required for preserving vision^1^. Although the anatomic success rate of retinal reattachment surgery by scleral buckling or pars plana vitrectomy (PPV) is high level, patients with RRD occasionally have incomplete recovery of their visual acuity^2–4^ because of permanent functional damage to the retina in the macular area by the detachment^5,6^. However, it is difficult to measure the functional damage before surgery. Therefore, it is still difficult for clinicians to predict the postoperative final vision accurately in eyes with macula-off RRD preoperatively.

Many preoperative factors such as age of the patient^4^, height of the macular detachment^7,8^, duration of the macular detachment^9,10^, and the preoperative best-corrected visual acuity (BCVA)^11^, have been reported to be factors influencing the recovery of the vision following reattachment of a macula-off RRD.

Optical coherence tomography (OCT) has been recently used to evaluate the retinal structure of eyes with a RRD in more detail. These assessments include the detection of small cystoid cavities in the inner nuclear layer (INL) and/or outer nuclear layer (ONL)^12^ and a dropout of photoreceptor back reflections^12–14^. Whether these structural changes are correlated with the postoperative visual acuity has not been determined^12–16^. In addition, many OCT studies have shown that the integrities of the hyperreflective retinal outer bands, e.g., the ellipsoid zone (EZ) and the external limiting membrane (ELM) were significantly correlated with the postoperative BCVA following the retinal reattachment^15,17–21^. A lengthening of the outer segments of the photoreceptors is frequently observed in eyes with a serous retinal detachment^22–25^ and with a RRD. However, there have been no reports on the relationship between the preoperative length of the photoreceptors and the postoperative BCVA in eyes after a successful reattachment of macula-off RRD.

There have been reports on the longitudinal evaluations of the changes in the retinal layer lengths at the same area in images obtained by SD-OCT, and the authors reported a significant increase in the length of several central retinal layers one month after the reattachment surgery^21,26,27^. It is believed that the distance between the ELM and the EZ is the inner segment (IS) length and that between the EZ and the retinal pigment epithelium (RPE) is the photoreceptor outer segment (OS) length. The postoperative lengths have been reported to be significantly correlated with the BCVA at 1 month following successful reattachment of a macula-off RRD^26^. However, there have been no reports on the association between the length of the preoperative photoreceptors and the postoperative BCVA and the integrity of the photoreceptor microstructures as determined by evaluations of the OCT images.

Thus, the purpose of this study was to determine the length of the preoperative photoreceptors in eyes with a macula-off RRD, and to determine whether the length was significantly correlated with the pre- and postoperative BCVA after successful reattachment of a macula-off RRD. In addition, we examined whether the length was significantly correlated with the postoperative integrity of the photoreceptor microstructures determined by SD-OCT.

## Results

### Demographics of patients

Five hundred and twelve eyes of 497 patients with macula-off RRD underwent scleral buckling surgery or pars plana vitrectomy (PPV) in the Department of Ophthalmology, Nagoya University between January 2013 to January 2017. Of these, 443 eyes were excluded for the following reasons; 22 had proliferative vitreoretinopathy grade C or worse, 41 had vitreous hemorrhage, 18 had a macular hole, 23 had diabetic retinopathy, 28 had postoperative development of dense cataracts, 17 had macular edema, 22 had a significant epiretinal membrane, 29 did not have suitable Spectralis OCT images for measurement before the surgery, 90 did not have suitable Spectralis OCT images before or after the surgery, and 153 had less than 12 months of follow-up period. In the end, 69 eyes of 69 patients with a macula-off RRD (mean age, 65.9 ± 9.7 years) were studied. The demographics and surgical parameters of the patients are shown in Table 1.

**Table 1:** Patient clinical characteristics.

Characteristic	Macula-off RRD
n (eyes)	69
Age (years)	46.6 ± 19.4
Male/female	46/23
Preoperative BCVA (logMAR)	0.58 ± 0.53
Axial length (mm)	25.2 ± 2.3
Duration of retinal detachment (days)	10.8 ± 10.9
Presence of PVD	36 (52%)
Presence of intraretinal cystoid cavities	51 (74%)
Drop out of backreflection	23 (33%)
Length of photoreceptor (µm)	102.8 ± 28.7
Height of subretinal fluid (µm)	400.2 ± 292.4
Scleral buckling/PPV(+PEA+IOL)	36/33

RRD = rhegmatogenous retinal detachment; BCVA = best-corrected visual acuity; LogMAR = logarithm of the minimum angle of resolution; PVD = posterior vitreous detachment; PPV = pars plana vitrectomy; PEA = phacoemulsification and aspiration; IOL = intraocular lens.

### Preoperative and Postoperative BCVA and OCT findings

**Figure 1:**
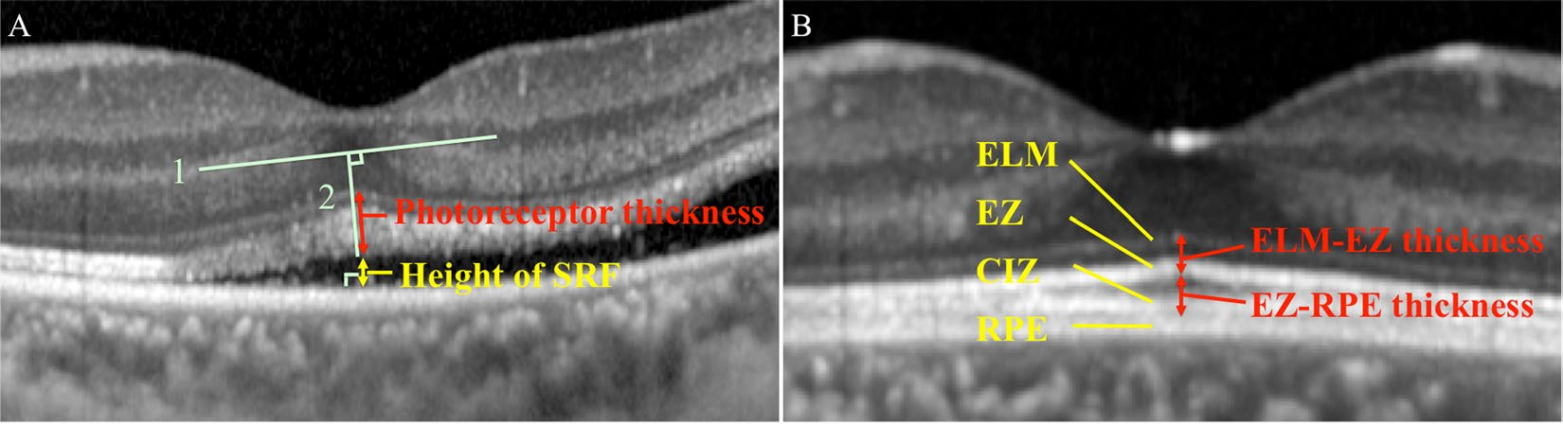
Measuring the preoperative photoreceptor length, the SRF height, and the postoperative length of the outer retinal layers. (**A**) The location of the foveal center was defined as the midway point between a straight line connecting the foveal terminations of the inner nuclear layers (Line 1). After drawing a straight line from the midpoint and orthogonal to Line 1 toward the outer retinal surface (Line 2), the length of preoperative photoreceptor was measured from the inner border of the external limiting membrane (ELM) and the outer end of foveal photoreceptors on Line 2. The height of the subfoveal fluid (SRF) was defined as the distance between the photoreceptor outer termination and the surface of the RPE along a straight line perpendicular to the surface of the retinal pigment epithelium (RPE). (**B**) ELM-ellipsoid zone (EZ) was measured as the distance between the ELM and the outer border of the EZ. EZ–RPE length was measured as the distance between the outer border of EZ and the inner border of RPE.

**Figure 2:**
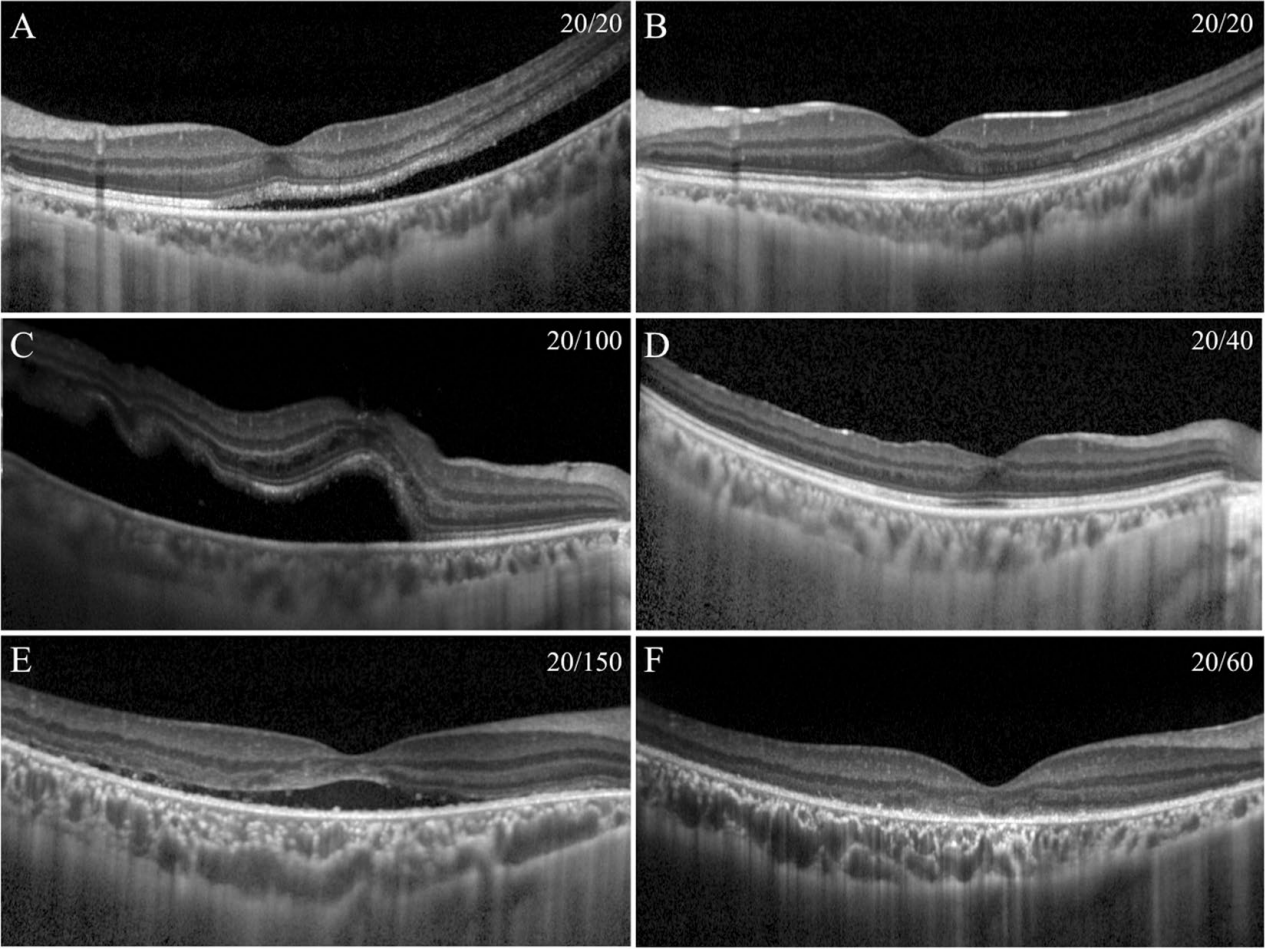
Representative spectral-domain optical coherence tomographic (SD-OCT) images of eyes with a RRD before and 1 year after the surgery. (**A**) The outer segments (OSs) of the photoreceptor were long in this 25-year-old man whose vision was 20/20. (**B**) One year after a successful re-attachment treated by scleral buckling procedures, the retinal outer bands and foveal bulge can be clearly seen and the vision was still 20/20. (**C**) The outer segments of photoreceptors were elongated, but cystoid cavities in the inner retina and a dropout of back reflections can be seen in a 39-year-old man whose vision was 20/100. (**D**) One year after successful re-attachment by scleral buckling procedures, the retinal outer bands were not so clearly observed, a foveal bulge was not present, and the vision was 20/40. (**E**) The outer segments of the photoreceptors were not seen in a 23-year-old woman who had a chronic RRD and vision was 20/150. (**F**) One year after successful reattachment treated by scleral buckling procedures, the retinal outer bands were disrupted and the vision was 20/60.

The preoperative BCVA was 0.58 ± 0.53 logarithm of the minimal angle of resolution (logMAR) units, and the postoperative BCVA was significantly improved to 0.12 ± 0.16 logMAR units (*P* < 0.001). The mean preoperative photoreceptor length was 102.8 ± 28.7 µm with a range of 20 to 159 µm (Fig. 1) and it was not correlated with the duration of retinal detachment (*r* = −0.183, *P* = 0.132). The mean height of the retinal detachment was 400 ± 292 µm. Preoperatively, 51 eyes (74%) had intraretinal cystoid cavities and 23 (33%) had a dropout of back reflections (Fig. 2).

Postoperatively, the mean IS length, i.e. the distance between the ELM and the EZ, was 30.0 ± 5.4 µm, the mean OS length, i.e. the distance between the EZ and the RPE was 36.2 ± 7.6 µm, and the mean photoreceptor length, i.e. the distance between the ELM and the RPE, was 66.2 ± 12.4 µm. The duration of the retinal detachment was significantly correlated with the postoperative photoreceptor length (*r* = −0.241, *P* = 0.046), but not significantly correlated with the preoperative photoreceptor length. The preoperative photoreceptor length was significantly longer than the postoperative photoreceptor length by approximately 1.5-fold (*P* < 0.001). The mean integrity score of the ELM was 3.3 ± 0.8, EZ was 3.2 ± 0.9, and the cone interdigitation zone (CIZ) was 2.5 ± 1.1.

### Correlations between preoperative BCVA and values of OCT parameter values

Univariate linear regression analysis showed that the preoperative BCVA was significantly correlated with the age (*r* = 0.247, *P* = 0.041), type of surgery (*r* = 0.258, *P* = 0.032), presence of a posterior vitreous detachment (*r* = 0.343, *P* = 0.004), presence of cystoid cavities (*r* = 0.313, *P* = 0.009), and height of the subretinal fluid (SRF; *r* = 0.563, *P* < 0.001; Table 2). The preoperative BCVA was not significantly correlated with the length of the preoperative photoreceptors (Fig. 1). Multivariate linear regression analyses revealed that the height of the SRF was a significant and independent factor associated with the preoperative BCVA (*β* = 0.536, *P* < 0.001).

**Table 2:** Univariate and multivariate regression analyses of the association between preoperative BCVA and clinical preoperative parameters in eyes with RRD.

Parameter	Univariate linear regression		Multivariate linear regression	
	*r*	*p*-value	*β*	*p*-value
Age	0.247	0.041	0.034	0.773
Sex	0.031	0.799	0.008	0.942
Surgical procedure	0.258	0.032	0.079	0.483
Presence of PVD	0.343	0.004	0.175	0.120
Axial length	−0.153	0.220	−0.052	0.642
Duration of detachment	−0.167	0.169	−0.062	0.573
Presence of cystoid cavity	0.313	0.009	0.126	0.274
Drop out of backreflection	−0.061	0.618	0.002	0.988
Length of photoreceptor	0.095	0.438	0.100	0.357
Height of subretinal fluid	0.563	<0.001	0.536	<0.001

BCVA = best corrected visual acuity; RRD = rhegmatogenous retinal detachment; PVD = posterior vitreous detachment.

### Correlation between preoperative OCT parameters and postoperative BCVA

**Figure 3:**
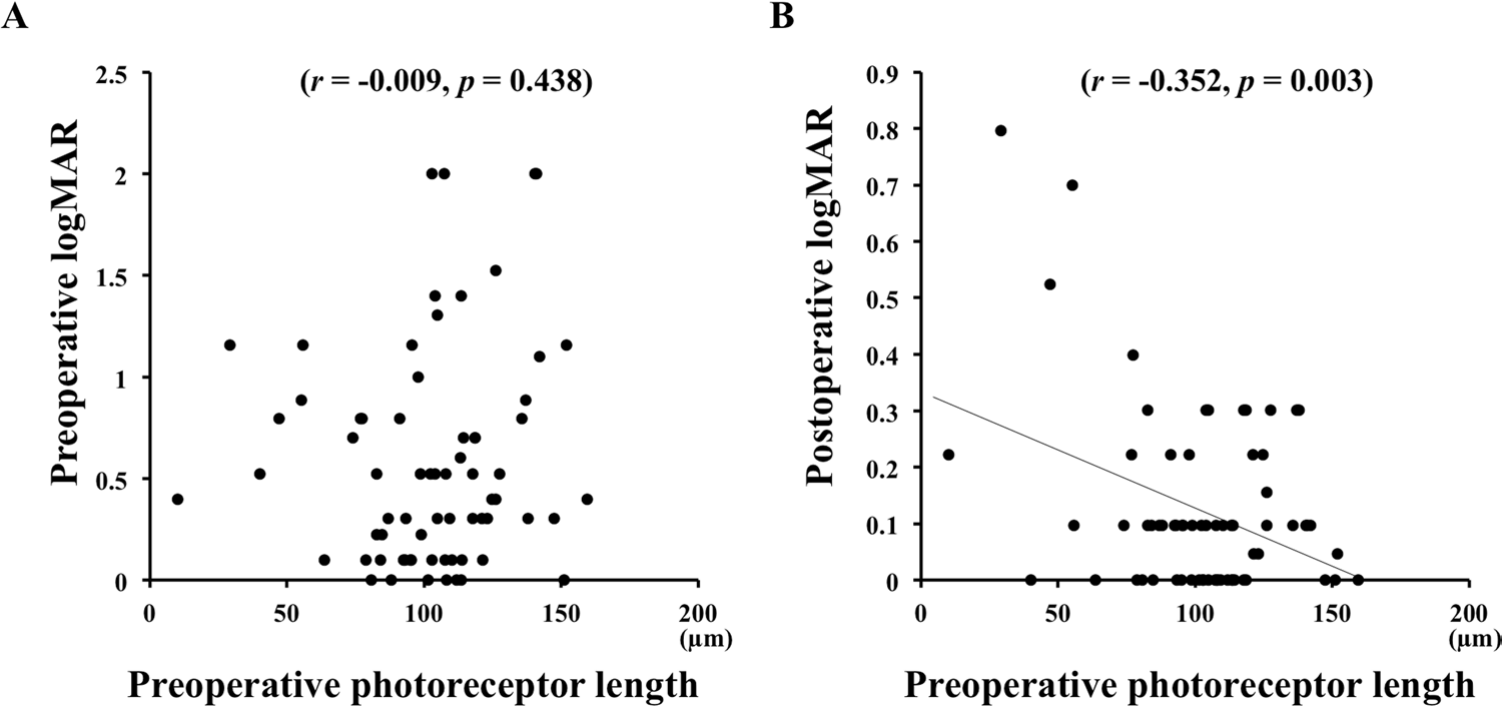
Correlations between the length of the preoperative photoreceptors and the preoperative BCVA (**A**) and the postoperative BCVA (**B**). The length of the preoperative photoreceptors was not significantly correlated with the preoperative BCVA but is significantly correlated with the postoperative BCVA (*r* = −0.352, *P* = 0.003). logMAR = logarithm of the minimum angle of resolution.

Univariate linear regression analysis showed that the preoperative photoreceptor length was significantly correlated with the postoperative BCVA (*r* = −0.353, *P* = 0.003; Table 3; Fig. 3). Multivariate linear regression analysis showed that the length of preoperative photoreceptor (*β* = −0.388, *P* = 0.001) and the preoperative BCVA (*β* = 0.274, *P* = 0.021) were significant and independent factors associated with the postoperative BCVA. The adjusted R^2^ value, which represent the amount of variability in the final vision estimation explained by the combined effect of the length of preoperative photoreceptor and the preoperative BCVA, was 0.181 in the multiple linear regression.

**Table 3:** Univariate and multivariate regression analyses of the association between postoperative BCVA and clinical preoperative parameters in eyes with RRD.

Parameter	Univariate linear regression		Multivariate linear regression	
	*r*	*p*-value	*β*	*p*-value
Age	0.072	0.559	0.079	0.515
Sex	0.118	0.335	0.138	0.250
Surgical procedure	0.029	0.815	0.024	0.842
Presence of PVD	−0.068	0.578	0.034	0.272
Axial length	−0.117	0.348	−0.102	0.214
Duration of detachment	0.201	0.097	0.152	0.775
Presence of cystoid cavity	−0.155	0.204	−0.059	0.641
Drop out of backreflection	0.169	0.166	0.088	0.487
Length of photoreceptor	−0.353	0.003	−0.388	0.001
Height of subretinal fluid	0.094	0.452	0.111	0.422
Preoperative BCVA	0.204	0.093	0.274	0.021

BCVA = best corrected visual acuity; RRD = rhegmatogenous retinal detachment; PVD = posterior vitreous detachment.

### Correlation between preoperative photoreceptor length and postoperative OCT findings

**Figure 4:**
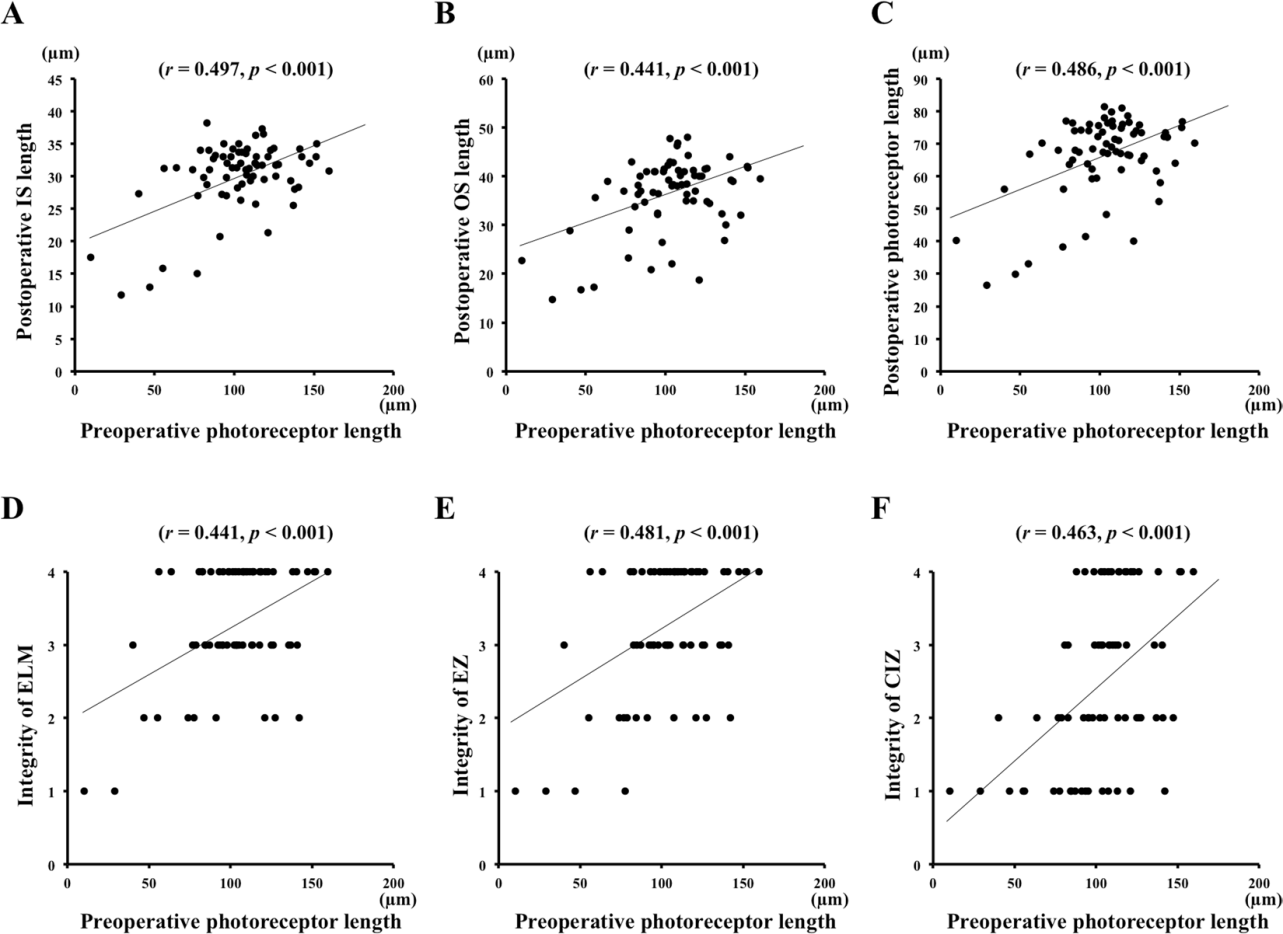
Correlations between the length of the preoperative photoreceptors and postoperative photoreceptors (**A**–**C**) and integrity of the microstructures of the photoreceptors (**D**–**F**). The length of the preoperative photoreceptors is significantly correlated with the postoperative photoreceptor inner segment (IS) length (*r* = 0.497, *P* < 0.001) (**A**), outer segment (OS) length (*r* = 0.441, *P* < 0.001) (**B**), the photoreceptor length (*r* = 0.486, *P* < 0.001) (**C**); integrity of the external limiting membrane (ELM) (*r* = 0.441, *P* < 0.001) (**D**), the ellipsoid zone (EZ; *r* = 0.481, *P* < 0.001)(**E**), and the cone interdigitation zone (CIZ). (*r* = 0.463, *P* < 0.001) (**F**).

Univariate linear regression analysis showed that the length of the preoperative photoreceptors was significantly correlated with the postoperative photoreceptor IS length (*r* = 0.497, *P* < 0.001), OS length (*r* = 0.441, *P* < 0.001), photoreceptor length (*r* = 0.486, *P* < 0.001), integrity of the ELM (*r* = 0.441, *P* < 0.001), integrity of the EZ (*r* = 0.481, *P* < 0.001), and the CIZ length (*r* = 0.463, *P* < 0.001; Fig. 4).

## Discussion

Our results showed that the length of the preoperative photoreceptors was significantly correlated with the postoperative BCVA after successful reattachment of a macula-off RRD. The length was also correlated with the postoperative outer retinal microstructures, viz., the photoreceptor length and the integrities of the foveal ELM, EZ, and CIZ microstructures. Although the preoperative height of the SRF was significantly correlated with the preoperative BCVA, it was not significantly correlated with the postoperative BCVA. Multiple regression analyses revealed that the length of the preoperative photoreceptors was an independent predictor of the final BCVA.

It is still not easy for surgeons to predict the postoperative BCVA accurately in eyes with macula-off RRD. Despite many factors have been found to influence the postoperative visual acuity in eyes with a macula-off RRD^4,7–10^, the most important predictor of the postoperative visual recovery after macula-off RRD surgery has been reported to be the preoperative BCVA^11,28–30^. In our study, the preoperative BCVA was significantly correlated with the final BCVA as reported, but the linear and multiple regression analyses revealed that the length of the preoperative photoreceptors had the highest correlation with the final BCVA.

Recent improvements of the resolution in OCT devices have enabled clinicians to obtain more accurate and precise evaluations of the retinal microstructures including quantitative analyses of the length of the different microstructures of the photoreceptors^21,26,27^. An elongation of the OSs of the photoreceptors has been reported in eyes with central serous chorioretinopathy (CSC)^22,23^ and Best disease^24^. Although similar findings have been reported for eyes with RRD, there have not been any studies that measured the length of the preoperative photoreceptor lengths in the OCT images of eyes with a RRD. This is probably because it is difficult to measure this length because the retina is detached and positioned diagonally to the surface of the RPE. In addition, the OCT images may not be clear due to vitreous hemorrhages and other opacities of the media in eyes with a RRD.

The length of the preoperative photoreceptors determined by SD-OCT was 1.5 fold longer than the postoperative photoreceptor length. Matsumoto *et al*. reported that the median length of the IS was approximately 35 µm in CSC eyes which was not significantly different from that of the normal eyes^22^. This finding indicates that the length of the IS was unchanged in eyes with CSC and only the length of the OS was elongated. The photoreceptor OSs are surrounded by the microvilli of the RPE in normal eyes, and they are continuously shed and phagocytosed by the RPE^31^. However, the photoreceptor OS at the sites of the detached retina should be elongated because of the lack of phagocytosis by the RPE. This may explain why the photoreceptor OS is longer in eyes with a detached retina and with a serous retinal detachment^22–24^.

The preoperative photoreceptor length ranged from 20 to 159 µm while the length of the postoperative photoreceptors has been reported to be 70–80 µm after successful retinal reattachment in eyes with RRD^21,26,27^. These findings indicate that the length of the preoperative photoreceptors was shortened in some eyes but was elongated in other eyes.

The sensory retina is detached from the RPE in both RRD and CSC eyes, but the conditions are different because the vitreous humor invades the subretinal space in eyes with a RRD. Kowalczuk *et al*. investigated the molecular composition of SRF in eyes with CSC and RRD by proteomics and metabolomics, and they reported that the SRF had better photoreceptor preservative properties in the CSC eyes^32^. In addition, it has been reported that macula-off RRD leads to a more rapid visual loss compared to eyes with CSC in spite of similar height of foveal detachment^12^. A disruption of the photoreceptor IS/OS in the foveal region was detected only in eye with RRD and not in eyes with CSC^13^.

Investigations on animal have shown that the photoreceptors under a detached retina undergo apoptosis^33–35^. Arroyo and associates obtained retinal tissue fragments during vitreous surgery for retinal detachment, and histological analyses showed that apoptosis occurred within 24 hours, peaked on day 2, and decreased to a low level after day 7 of a retinal detachment^35^. Experimental studies have shown a loss of the OS of the photoreceptors after a detachment of the photoreceptors from the RPE, whereby disrupting the normal OS renewal and leading to a shortening of the OSs and eventual degeneration of the photoreceptors^36–38^. Various changes develop in several cell types throughout the retina that is caused by separation of the neural retina from the RPE. These findings indicate that the photoreceptor OSs should drop out or at least shorten with time after a retinal detachment.

Our results showed that the length of preoperative photoreceptors was significantly correlated with the length of the postoperative photoreceptors and the integrity of foveal ELM, EZ, and CIZ microstructures. In addition, multivariate linear regression analyses revealed that the length of preoperative photoreceptor and the preoperative BCVA were significant and independent factors associated with the postoperative BCVA. However, if the factor “length of preoperative photoreceptor” was not used in multivariate linear regression analyses, no variables were found to be significantly associated with the postoperative BCVA (not shown). It has been reported that the length of the outer retinal layers increases and the integrity of outer retina recovers after successful retinal reattachment, and these changes are correlated with an improvement of the BCVA^21^. Many SD-OCT studies have demonstrated that the integrity of the retinal microstructures is significantly correlated with the BCVA following retinal reattachment^15,17–21,39^. Taken together, the detachment of the sensory retina from the RPE causes apoptosis of the photoreceptors which results in a shortening of the OS of the photoreceptors. This then leads to a decrease in the BCVA. Eyes with severe retinal damage with apoptosis before surgery have shorter preoperative photoreceptors and poorer integrity of the microstructure of the foveal photoreceptors resulting in a poorer postoperative final BCVA. On the other hand, eyes with not too severe retinal damage with apoptosis before surgery, such as those with small retinal tears and with slower progression of the retinal detachment have longer photoreceptors before the surgery, longer postoperative photoreceptors, and better integrity of the foveal photoreceptors all of which result in a better final BCVA. Accordingly, the length of the preoperative photoreceptors can be a good factor for a predictor for the recovery of the BCVA.

Earlier studies reported that the duration of the retinal detachment was correlated with the visual recovery^9,10^. However, our result did not find a significant correlation between the duration of retinal detachment and the pre- and postoperative BCVA. One possibility for this discrepancy is that the characteristics of the SRF were different among the eyes with RRD even though the duration of the detachment was similar. In eyes with a single small retinal break, especially in younger patients, a smaller volume of vitreous humor would flow into the subretinal space. Then, the photoreceptor cell apoptosis would progress more slowly which may cause a similar situation as eyes with CSC. In such situations, even if the duration of the retinal detachment is longer, the apoptosis would not progress rapidly, and the photoreceptor would be longer, and the preoperative vision would not decrease significantly. Under these conditions, the vision can be restored after successful reattachment, and the duration of retinal detachment might be not correlated with the final BCVA.

This study has several limitations. First, this was a retrospective study on a relatively small sample which would lower the statistical power of the analyses. Second, eyes treated by both of scleral buckling and PPV were included. The postoperative time course determined by OCT between eyes treated by scleral buckling and PPV surgeries should be different because the SRF tends to remain in eyes treated with scleral buckling. However, none of the eyes had SRF in the OCT images after 12 months when we were able to evaluate the OCT findings more accurately. Third, we measured the length of the preoperative photoreceptor from the inner border of ELM to the end of the outer segment of foveal photoreceptor in the OCT images. This was done because the EZ layer was not clear and we could not divide the photoreceptor into ISs and OSs in the preoperative OCT images. Fourth, the effect of the size of the retinal tear was not evaluated. The size of retinal break might be correlated with the length of preoperative photoreceptor. Fifth, we performed vitrectomy combined with cataract surgery for some patients, but eyes with severe cataract formation were excluded. However, the vision improvements by cataract surgery should be slight. Further prospective studies on a larger number of eyes with automated calculations on retinal lengths will be necessary to validate that a preoperative longer photoreceptors can be a predictor of the BCVA at one year.

In conclusion, the BCVA improved significantly following successful retinal reattachment. A longer length of the preoperative photoreceptor is significantly correlated with longer postoperative photoreceptors with better integrity of the outer photoreceptor microstructures after successful reattachment. The length of the preoperative photoreceptors can be a good candidate for predicting the final BCVA following RRD surgery in eyes with a macula-off RRD.

## Patients and Methods

### Ethics statement

The procedures used in this observational, single-center study conformed to the tenets of the Declaration of Helsinki, and they were approved by the Institutional Review Board and Ethics Committee of the Nagoya University Graduate School of Medicine. A signed written informed consent was obtained from all patients.

### Subjects

We reviewed the medical records of all patients who had undergone successful RRD repair by vitrectomy or scleral buckling at the Nagoya University Hospital from January 2013 to August 2017. All patients had a retinal detachment involving the macula, i.e., a macula-off RRD, and they were evaluated by SD-OCT macular scans pre- and postoperatively.

All patients had comprehensive ophthalmic examinations including measurements of the BCVA, intraocular pressure, and axial length. They also had slit-lamp, and ophthalmoscopic and SD-OCT examinations before and at least 12 months following the surgery. The Snellen VA values were converted to the logMAR units for the statistical analyses.

### Surgical techniques

Retinal detachment surgery was performed with a conventional scleral buckling procedure or PPV. For the scleral buckling surgery, the retinal breaks were identified and treated by transscleral cryotherapy. Mattress sutures were placed 7.0 to 7.5 mm apart with 4–0 supramid (Kono, Chiba, Japan) for the circumferential segmental buckle and a silicone sponge (Mira No. 506; Mira, Inc, Waltham, MA) was sutured as an explant in all cases. The SRF was drained if necessary. An intraocular tamponade was not used in all cases.

For the PPV surgery, after creating the three ports, PPV was performed using a 23- or 25-gauge Constellation® system (Alcon Laboratories, Inc., Fort Worth, TX) as described in detail^21^. The vitreous was removed as completely as possible, then fluid-air exchange and subretinal fluid drainage from the causative retinal tear(s) or iatrogenic hole were performed. Endo-photocoagulation was applied to the causative retinal tear(s) or any iatrogenic holes. Then 20% sulfur hexafluoride (SF6) was injected into the vitreous upon completion of the PPV. Cataract surgery with phacoemulsification and intraocular lens implantation were performed at the time of PPV if the cataract was visually significant or if it was deemed necessary for successful reattachment of RRD.

### Measurements of photoreceptor microstructures in OCT images

A SD-OCT instrument (Spectralis^®^, Heidelberg Engineering, Heidelberg, Germany) was used to obtain cross-sectional images of the retina in all cases. We evaluated six radial cross-sectional images recorded at each visit before and after the successful retinal reattachment surgery.

The preoperative OCT analysis included back reflection which was defined as smudges of the ELM, the photoreceptor IS/OS junction, or the EZ^12–14^, height of the SRF at the fovea, intraretinal cystic cavities in the INL and/or ONL, and the length of photoreceptor (Fig. 1). The height of the SRF at the fovea was measured manually as the distance between the inner border of the retinal pigment epithelium and the outer border of the back reflection with the caliper embedded in the Spectralis software. The length of the photoreceptors was also measured manually as the distance from the inner border of ELM to the outer termination of the foveal photoreceptors. A dropout of the IS/OS of the foveal photoreceptors was defined as defects of back reflection that are seen beneath and in contact with the highly reflective line representing the ELM.

The preoperative OCT analysis included measurements of the length of the different retinal layers in the OCT images as described in detail^21,25^. The ELM-EZ length which is equivalent to the length of the IS of the photoreceptors was defined as the distance between the outer borders of ELM to the EZ, and the EZ-RPE length which is equivalent to the length of the OSs of the photoreceptors was defined as the distance between the outer border of EZ and the inner border of RPE^40^. The postoperative photoreceptor length was defined as the distance between the outer border of the ELM and the inner border of the RPE. The postoperative integrity of the foveal ELM, EZ, and CIZ was determined for a 1-mm-diameter area of each image and quantified on a 4-point scale: 1, line not visible; 2, line disruption >200 μm; 3, line disruption <200 μm; and 4, continuous line. Identical measurements were performed on the fellow eyes as controls^25^.

### Exclusion criteria

Eyes were excluded if; presence of ocular media opacities, e.g., severe cataract, vitreous hemorrhage, vitreous opacity, macular abnormalities, e.g., macular hole, vascular occlusive diseases, or diabetic retinopathy, were present, proliferative vitreoretinopathy (PVR) more severe than grade C^41^, and clinically evident postoperative changes that were likely to interfere with accurate evaluations of the retinal layers, e.g., recurrent RRD, epiretinal membrane, cystoid macular edema, or persistent SRF, were present.

### Statistical analyses

The values are presented as the means ± standard deviations. Independent *t* tests were used to determine the significance of the differences of normally distributed data and the Chi-square test for the categorical data. Pearson’s correlation coefficient tests were used to determine the significance of the associations between each parameter and the BCVA. Multiple linear regression analyses were used to evaluate the association between the preoperative and final BCVA and the preoperative independent variables.
